# Mast Cells Accumulate in the Stroma of Breast Adenocarcinoma and Secrete Pro-Inflammatory Cytokines and Tumor-Damaging Mediators: Could IL-37 and IL-38 Play an Anti-Tumor Role?

**DOI:** 10.3390/ijms27020824

**Published:** 2026-01-14

**Authors:** Pio Conti, Carla E. Gallenga, Ciro Annicchiarico, Armando Coppola, Raffaello Pellegrino, Michelangelo J. Conti, Filiberto Mastrangelo

**Affiliations:** 1 Immunology Division, Postgraduate Medical School, University of Chieti, 66100 Chieti, Italy; 2 Section of Ophthalmology, Department of Biomedical Sciences and Specialist Surgery, University of Ferrara, 44121 Ferrara, Italy; 3 Independent Researcher, 74023 Grottaglie, Italy; 4 Private Practice, Studio Armando Coppola, Corso Garibaldi 246, 80141 Naples, Italy; 5 Independent Researcher, Lecce-Castromediano/Cavallino, Via S. Pelico 34, 73100 Lecce, Italy; 6 Faculty of Medicine, S. Andrea University “La Sapienza”, 00189 Rome, Italy; 7 Department of Clinical and Experimental Medicine, School of Dentistry, University of Foggia, 71100 Foggia, Italy

**Keywords:** cancer, mast cell, adenocarcinoma, inflammatory cytokine, anti-inflammatory cytokine

## Abstract

Tumor tissue is surrounded by mast cells (MCs), which participate in the inflammatory immune response by producing cytokines, proteases, and other molecules. MCs are involved in both innate and acquired immunity and are associated with the IgE response through the FcεRI receptor. MCs mediate inflammation in several immune reactions, including acute hyperreactivity, leukocyte recruitment, acute tissue swelling, anaphylaxis, and pro-inflammatory cytokine production. They not only function as pro-inflammatory effector cells but may also contribute to the regulation of the acquired immune response in tumor tissue. Therefore, MCs may mediate immunity in breast cancer by promoting remodelling and counteracting cancer growth. They also produce anti-inflammatory substances, such as histamine, transforming growth factor-β (TGF-β)1, IL-10, and IL-4, which inhibit the acquired immune response and reduce the inflammatory state. IL-37 and IL-38 are novel natural regulators of inflammation and are anti-inflammatory members of the IL-1 family. IL-1, generated by immune cells such as macrophages and lymphocytes, is released downstream of oncogenes in breast cancer, triggering an inflammatory response by stimulating other pro-inflammatory cytokines such as IL-6, tumor necrosis factor (TNF), and IL-33 (an early warning cytokine). Therefore, blocking IL-1 with IL-37 or IL-38 could represent a novel therapeutic strategy that, when combined with other treatments, could be beneficial in breast cancer. This review focuses on the new discoveries and insights into the role of MCs in breast cancer. We also analyzed molecules that can promote tumor growth and those that can inhibit cancer development and metastasis. This review aims to study the role of MCs accumulated in the stroma of breast adenocarcinoma in relation to secreted anti-inflammatory cytokines, such as IL-37 and IL-38.

## 1. Introduction

Repair and tolerance keep DNA stable, but dysregulation is one of the causes of tumor onset [1]. Cancer is a non-hereditary disease, but some individuals may have a genetic predisposition to develop certain types of tumors. Genetic mutations in DNA can lead to cancer, which may be caused by physical, chemical, biological, or hormonal factors [2].

Breast cancer is prevalent in women, and despite the improvement of therapeutic targets in the last 20 years, its incidence and fatality have dramatically increased [3]. High estrogen levels and environmental factors are often associated with the risk of breast cancer. Elevated blood levels of androgens, which can be converted to estrogen and estradiol, can also cause breast cancer [4,5]. Each year in the United States, about 300,000 cases of breast cancer are diagnosed in women, and about 40,000 women die from this disease [6].

In the middle of the last century, patients with breast cancer had poor survival results; however, today, the situation has greatly improved, with greater therapeutic efficacy, a higher degree of recovery, and increased quality of life [7]. However, some breast cancers are still resistant to immunotherapy against tumor antigens, despite the wide knowledge of antigen mechanisms of action. The improvement of tumor immunity and the advancing knowledge of the inflammatory processes occurring around the tumor mass will lead to more effective therapies and an increase in life span for breast cancer patients [8].

Breast adenocarcinoma, originating from the glandular epithelium of the ductal-lobular units, promotes a pro-inflammatory and immunosuppressive microenvironment. It recruits M2 macrophages, Tregs, and myeloid suppressor cells, which generate IL-1, promote tumor growth, and exert immune evasion via programmed death-ligand 1 (PD-L1).

IL-37 exerts direct anti-inflammatory action and blocks nuclear factor kappa B (NF-κB) and mitogen-activated protein kinase (MAPK), reducing tumor growth, angiogenesis, and metastasis. IL-38 regulates the IL-36/IL-17 axis by modulating inflammation, possibly reducing tumor activity. Both new cytokines, IL-37 and IL-38, block IL-1.

Mast cells (MCs) are highly plastic cells in breast cancer that can activate innate and adaptive responses, support angiogenesis, and participate in invasiveness and stromal remodelling. Their function is context-dependent and represents a potential therapeutic target for immune modulation. IL-37/IL-38 can inhibit the pro-tumor activity of infiltrating MCs and macrophages by reducing angiogenesis and inflammation and inhibiting the cancerous microenvironment. Inhibition of IL-1 production by MCs and macrophages may represent a potential therapeutic target for modulating the microenvironment in breast adenocarcinoma.

Tumor cells suppress the immune response by inhibiting T regulatory (Treg) cells and increasing T helper cell (Th)1 activation. The anti-inflammatory cytokine IL-10, expressed by immune cells, including MCs, is also suppressed by breast cancer cells [8,9]. MCs secrete IL-10 upon stimulation with bacterial products, such as lipopolysaccharide (LPS). IL-10 is an immunoregulatory and inflammatory inhibitor cytokine secreted by several cells, such as T cells, B cells, dendritic cells, macrophages, natural killer (NK) cells, eosinophils, neutrophils, Th1, Th2, and Th17 cells [10]. Since IL-10 can limit the severity of severe cutaneous reactions by inhibiting inflammation in *kit^w/w-v^* mice, it is pertinent to consider that this cytokine has a negative effect on malignant epithelial cell proliferation, such as that in breast cancer [11]. The most important mechanism of IL-10 production by MCs is established via IgG1 and the FcγRIII receptor, which is more important than the activation of MCs with IgE binding to FcεRI [12]. These immune responses occur in the immune cells surrounding the tumor tissue and in lymph nodes infiltrated by tumor cells, where mature CD83^+^ dendritic cells are fewer in number compared to non-infiltrated lymph nodes. Lymph nodes are not directly involved in the tumor process, meaning they are not infiltrated by tumor cells and are therefore considered metastasis-negative. CD83^+^ mature dendritic cells in lymph nodes decrease due to alteration and suppression of the immune system in the microenvironment, caused by mediators such as IL-10 and transforming growth factor-β (TGF-β). This demonstrates that lymph nodes are not involved in the tumor process and can maintain a certain immune response [9].

In the breast cancer microenvironment, an angiogenic process occurs among tumor cells, with new blood vessel formation and the presence of various cell types, including fibroblasts, stem cells, and white blood immune cells [13]. In tumor dynamics, new antigens are generated that encounter immune cells, such as antigen-presenting cells (APCs), that activate cytotoxic CD8^+^ T and CD4^+^ T lymphocytes, although this reaction is not efficient in suppressing tumor cells [14]. CD8^+^ cells, which increase in the tumor, have a cytolytic action with the release of molecules such as granzyme and perforin that mediate inflammatory and apoptotic processes [15]. Inflammation from local immune reactions is a characteristic process of breast tumor proliferation [16]. Inflammatory cytokines and other mediators produced by both tumor cells and infiltrated immune cells, such as chemokines, transcription factors, acute phase proteins, histamine, vascular endothelial growth factor (VEGF), and proteases, are present at the inflamed tumor site, and anti-inflammatory proteins may provide relief from side effects, including pain and inflammation [17]. The tumor necrosis factor (TNF) ligand, which is generated by stromal cells, tumor-associated macrophages, endothelial cells, and cancer cells, has anti-tumor effects and amplifies the action of CD8^+^ and CD4^+^ T cells, inducing apoptosis and inflammation [18].

MCs accumulate in the tumor stroma of human breast cancer, where they increase in number and carry out their biological activities by participating in angiogenesis, cell proliferation, metastasis, and invasion in the tumor microenvironment. However, the mechanisms underlying the involvement of MCs in breast cancer tissue remain unclear [19].

## 2. Mast Cells (MCs)

MCs are derived from myeloid bone marrow lineages, CD34^+^/CD117^+^/CD13^+^, and are involved in immune and inflammatory reactions and tissue homeostasis in many diseases, including cancer [20] (Table 1). After their discovery, it was highlighted that they could mediate allergic reactions; however, these cells were later shown to be capable of mediating acute and chronic inflammation, tissue repair, angiogenesis, and tumors.

MCs develop through their proto-oncogenic transmembrane receptor tyrosine kinase growth factor *c-kit*/CD117 (where *c-kit* is the gene and CD117 is the protein), which is also present in other cell types. The CD117 protein, which is present on MC membranes, has been found to be a marker for some tumors [12]. In mouse experiments, *c-kit* expression was found in immature germ cells. These useful observations were obtained from the study of *KitW-f/KitW-f*, *KitW/KitW-v*, and *KitW-sh/KitW-sh* mice, which show dysregulation of c-kit genes.

MC activation occurs after receptor-ligand binding and results in increased intracellular calcium flux, leading to the mediation of various diseases, such as cancer. IgE antibodies bound to the cellular high-affinity receptor FcεRI cause aggregation of the receptor with biological responses. This reaction triggers a biochemical cascade, leading to the generation of inflammatory compounds [12].

Upon activation, MCs produce various compounds, including IgE and pro-inflammatory cytokines [17]. When IgE binds to FcεRI, it leads to the immediate release (after a few seconds) of compounds such as histamine, TNF, and proteases [21]. Cytokines such as IL-1 activate MCs, leading to the de novo synthesis of pro-inflammatory cytokines, including IL-1, TNF, IL-6, and IL-33 [22].

Therefore, MCs are involved in many physiological and pathological processes in the body and play an important role as effector cells in IgE-mediated allergic responses, inflammation, and T cell-mediated immunity. MCs communicate with surrounding tissues and often with the external environment. Upon activation, they produce a wide range of inflammatory mediators that can be quickly released after activation or delayed through mRNA production (Table 2). Moreover, certain stimuli, including cancer, can activate MC phospholipases, causing the production of arachidonic acid compounds, such as prostaglandin D2 (PGD2) and leukotrienes, which mediate inflammation and pain [12]. MCs are mediators of both innate and acquired immunity and can be activated by a number of stimulators, such as peptides, drugs, cytokines, growth factors, and environmental factors (Table 3).

## 3. The Pro and Anti-Tumor Roles of MCs

MCs have hormone receptors and are more abundant in malignant tissues [23]. Along with breast cancer cells, they express c-kit receptors, which can be a negative diagnostic sign for survival when missing. Furthermore, MCs play an important role in the development of metastases, and experiments on mice have determined that animals deficient in MCs develop fewer tumors and have fewer metastases [24]. In breast tumor tissue, MCs can be recruited by chemokines such as monocyte chemoattractant protein-1 (MCP-1/CCL2) and regulated on activation, normal T cell expressed and secreted (RANTES), as well as by the cytokine colony-stimulating factor (CSF) [8]. This would allow MCs to selectively secrete beneficial (friendly) substances for cancer. For example, histamine, a mitogenic compound, can act as a growth factor and immune suppressor, while VEGF and IL-8 participate in angiogenesis. Tumor growth can be mediated by stem cell factor (SCF), nerve growth factor (NGF), and platelet-derived growth factor (PDGF) [12]. However, MCs can also be detrimental to tumors (foes) by producing apoptosis-inducing cytokines such as IL-4 and TNF. In contrast, IL-6, IL-1, and IL-33 can inhibit metastasis and mediate breast cancer cell death [8].

IL-1, which is generated downstream of oncogenes, causes chronic inflammation in breast cancer, resulting in an important mediator [25]. Chronic inflammation can cause tissue damage, which can lead to tumor progression. IL-1 drives inflammation in the tumor microenvironment via the NF-κB pathway through the activation and generation of other cytokines and chemokines. Although being an important immune molecule for lymphocyte activation, IL-1 can have an anti-tumor effect. On the other hand, it is a potent inflammatory cytokine that mediates tumor formation, including breast cancer [26].

**Figure 1:**
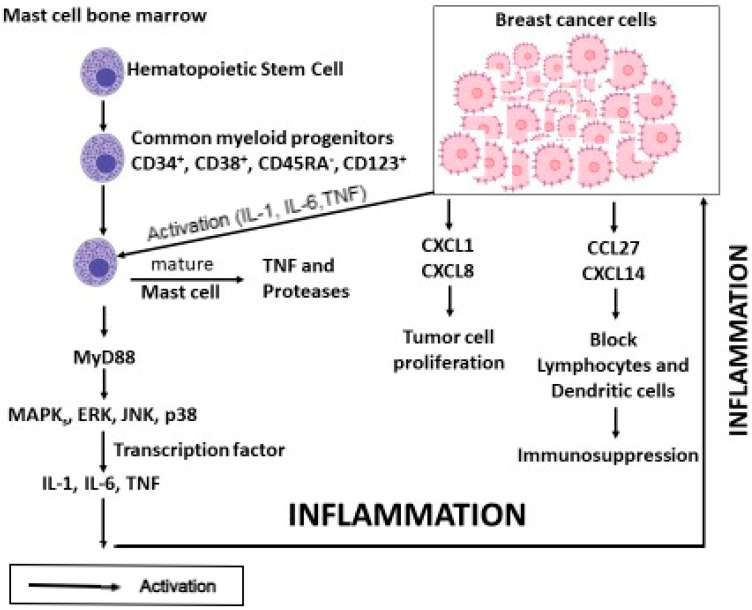
Mast cells (MCs) are derived from the bone marrow and are activated in breast cancer by signals released in the tumor microenvironment, such as IL-1, IL-6, tumor necrosis factor (TNF), *c-Kit* ligand, IL-33, and others. Once activated through the MyD88 pathway, MCs produce pro-inflammatory cytokines, such as IL-1, IL-6, and TNF, which induce inflammation. TNF can be produced by MCs through both degranulation and mRNA. In addition, tumor cells release the chemokines CXCL1 and CXCL8, which are involved in tumor cell proliferation, while the chemokines CCL27 and CXCL14 produced by tumor cells cause immunosuppression by blocking lymphocytes and dendritic cells.

MCs are derived from common progenitor cells and are activated in breast cancer angiogenesis, where they generate TNF, proteases, IL-1, and IL-6, which induce inflammation. In contrast, chemokines CXCL1 and CXCL2 participate in tumor cell proliferation, whereas CCL27 and CXCL14 cause immunosuppression by blocking lymphocytes and dendritic cells [8] (Figure 1).

Furthermore, in breast cancer, the ligand CCL21 participates in the recruitment of type 3 innate lymphoid cells (ILC3), which promotes the production of CXCL13 chemokines in stromal cells that regulate stromal ILC3 [27].

IL-1 drives the inflammatory response in cancer by affecting all components of the vessel wall [28]. Moreover, it can activate endothelial and smooth muscle cells, inducing certain chemokines, such as CCL2 and prostaglandins [29]. In breast cancer, IL-1 binds to its receptor IL-1R, activates the NOD-like receptor family pyrin domain containing (NLRP)3 inflammasome, and induces IL-6 along with IL-1, which mediates the acute phase response and fever in cancer patients [30].

Inflammasomes are activated by a pattern-recognition receptor, the adapter protein, and caspase-1, to process IL-1β in response to damage-associated signals [26]. Inflammasome activators, which are complex high-molecular-weight molecules, cause the secretion of caspase-1, mediating mature IL-1β in immune system cells [27]. Several inflammasomes containing different proteins have been described, including NLRP1, NLRP3, NLRP6, NLRP12, and others [31]. Upon binding to the inflammasome, caspase-1 is cleaved and activated, leading to the cleavage of several targets and provoking the maturation and secretion of inflammatory IL-1β [28]. Inflammasomes can be activated in MCs and macrophages through different signals, including pathogen-associated molecular patterns (PAMPs) and endogenous danger signals [32]. Inflammasome activation leads to an inflammatory response that can be beneficial or detrimental to the tumor. Accumulating evidence suggests that in breast cancer, there is harmful sterile inflammation caused by host-derived cancer cells [8].

Scientific data suggest that MCs are located around tumor masses and are involved in the development of tumor resistance [33]. They can participate in tumor rejection by producing molecules against cancer development and can also promote tumor growth by participating in angiogenesis, degradation through proteases, and immune response suppression. Angiogenesis is promoted by MCs through the synthesis of VEGF, proteases, TGF-β, NGF, and chemokines [33]. MC products can stimulate other inflammatory cells in the cancer cell microenvironment, cooperating with them in angiogenesis and tissue remodelling [12]. MCs release the chemokines MCP-1, RANTES, and SCF that cause the recruitment of immune cells such as eosinophils, neutrophils, and T and B lymphocytes [34]. MCs can play an autocrine role by recruiting themselves and secreting mitogenic and immunosuppressant factors, such as histamine, heparin, VEGF, and proteases, which promote the formation of new vessels and metastases, which can be beneficial to the tumor [8].

Regarding the production of TNF by MCs, both TNF stored in the granules and that produced through protein synthesis stimulate the production of other cytokines, such as IL-1 and IL-6, inducing apoptosis and counteracting tumor growth [12]. Additionally, the release of chondroitin sulfate and tryptase could oppose the development of breast cancer by inhibiting the formation of metastases [8]. MC metalloproteinases also participate in the degradation of tumor stroma with the release of angiogenic molecules, and the angiogenic process in breast cancer increases the vascular supply, which is involved in metastatic activity [35].

Tyrosine kinase receptors, including KIT (CD117), are a family of receptor proteins that are upregulated in tumor cells, and their mutations are associated with several stromal tumors. MCs express high levels of c-kit (involved in development, survival, and migration) and its ligand SCF, which enhances tumor growth by stimulating cytokines such as VEGF, TNF, IL-6, and IL-1 [17].

Members of the IL-1 family participate in cancer progression and stimulate other pro-inflammatory cytokines, such as IL-18, which stimulates interferon gamma (IFN-γ) in several cells, including NK cells and type 1 innate lymphoid cells (ILC1) [25]. IFN-γ opposes cancer development and improves patient prognosis [26].

Cancer therapy includes different approaches, such as surgery, radiation, chemotherapy, and biological therapy. The latter involves the use of immune molecules such as monoclonal antibodies, cytokines (TNF, IL-2, IFNs, granulocyte-macrophage colony-stimulating factor (GM-CSF), granulocyte colony-stimulating factor (G-CSF), etc.), DNA polymerase methylation inhibitors, cancer suppressor gene products, stem cells, and bone marrow transplantation, among others [36]. These therapies have limited efficacy; therefore, new strategies are needed to combat cancer.

MCs can express and activate a cytoplasmic protein complex, the NLRP3 inflammasome, in response to antigens such as toxins, allergens, and extracellular ATP, leading to the production of IL-1β. Inflammasome activation can lead to the release of IL-33, although IL-33 does not require caspase-1 processing like IL-1β, and the inflammasome can trigger the release of IL-33. The inflammasome can cause cell death and synthesize IL-33.

IL-33 is an early warning cytokine secreted and released by MCs that regulates the immune system. IL-33 strongly activates MCs, which release mediators that promote the growth, vascularization, and progression of breast cancer. The IL-33/MC pathway is considered a tumor driver.

## 4. IL-33

IL-33 is a cytokine that is also called “alarmin” because it is secreted as a warning signal by damaged or necrotic cells that participate in crosstalk with the immune system [37]. The mammalian IL-33 protein is an evolutionarily conserved member of the IL-1 family and is more similar to IL-18 than to other cytokines of the same family. At the gene expression level, the human IL-33 gene is located on the short arm of chromosome 9 at position *9p24.1*, while the mouse IL-33 gene is located on chromosome *19q24.1* in region 1/4 [30].

IL-33 carries out its biological activity by binding to its suppression of tumorigenicity 2 (ST2/IL-1R4) receptor and its co-receptor accessory protein (IL-1RAcP) [38]. IL-33 is a mediator of type 2 innate and adaptive immunity that acts on M2, type 2 innate lymphoid cells (ILC2), and Th2 macrophage responses in tissue repair and allergic phenomena [39]. It is a nuclear protein, also called nuclear factor from high endothelial venules, and it recruits the IL-1RAcP co-receptor by binding to its ST2 receptor, causing the intracellular activation of lymphoid cells.

Bone marrow MC stem cells are an important source and producer of IL-33 [39]. Certain MCs, such as MC chymase, can degrade active IL-33, rendering it inactive [40]. IL-33 modulates the immune response of several cells, including ILC2, Th2, and M2 macrophages, and can be derived from tumor cells, with the ability to recruit TGF-β, a cytokine that has pro-tumor activity by suppressing the activity of cytotoxic T cells (CTLs) [41].

**Figure 2:**
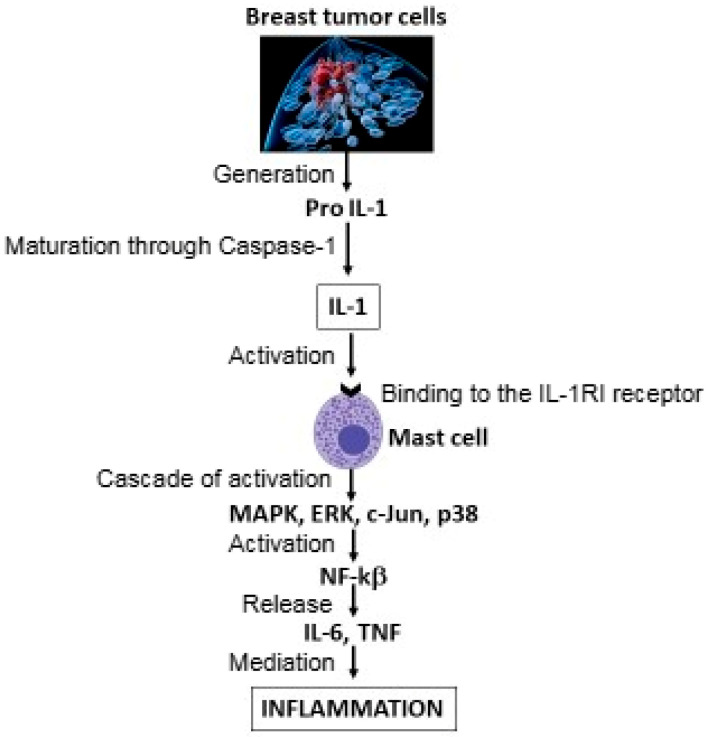
Breast cancer cells generate pro-IL-1, which is activated by caspase-1. IL-1 binds to the IL-1RI receptor on mast cells (MCs), causing a cascade of activation of mitogen-activated protein kinase (MAPK), extracellular signal-regulated kinase (ERK), cJun, and nuclear factor kappa B (NF-κB), with the subsequent release of IL-6 and tumor necrosis factor (TNF), which mediate inflammation. This figure shows how tumor cells can produce IL-1, which activates other inflammatory cytokines (IL-6 and TNF).

IL-1 is secreted by MCs and is involved in immunoregulation and inflammation. The immune system is responsible for immunosurveillance and is closely related to tumor diseases, including breast cancer. Cytokines are known to be involved in the advancement of tumor cell proliferation; however, some may be inhibitory, while others are stimulatory. Pathogens can activate NF-κB, which results in the production of pro-IL-1 and activation of the inflammasome (NLRP3), leading to the release of mature IL-1 from various cells, such as epithelial cells, myeloid cells, fibroblasts, dendritic cells, and mononuclear cells [42] (Figure 2).

IL-1 plays a central role and is a key mediator of the host response in various immunological and cancerous diseases. In breast cancer, IL-1 appears to be involved in the cytotoxicity and killing of host tumor cells [43]. Macrophages from cancer patients incubated in vitro produced less IL-1 than those from healthy patients. When recombinant IL-1 is incubated in vitro in the presence of other cytokines, such as IFN and IL-2, it increases the cancer cell killing capacity [43]. Therefore, IL-1 proves to be an anti-tumor immune cytokine; in fact, patients affected by cancer, who are immune depressed, have reduced production of IL-1.

In contrast, IL-1 is also a molecule that induces acute phase proteins (APPs) in the liver, such as serum amyloid A (SAA), which inhibits NK cell activity. Thus, IL-1 is a pro-tumor cytokine and seems to be an important molecule that favors tumor development.

IL-1 is involved in the stimulation of nitric oxide (NO) and reactive oxygen species (ROS), which are known to cause DNA damage [44]. In these reactions, the organism implements a defensive DNA repair system through the production of IL-22, which proves to be an anti-tumor cytokine [45].

IL-33 is involved in the regulation of infectious and inflammatory diseases, and upon activation, it is secreted by MCs, ILC2, and Treg cells. In addition, it stimulates cytokines IL-4, IL-5, and IL-13, and ILC2 cells are involved in allergic phenomena and autoimmune responses [46]. In rodent experiments, IL-33 deficiency has been shown to cause an allergy-like reaction, demonstrating the efficacy of this cytokine in the adaptive immune response [46]. When overexpressed, nuclear IL-33 can inhibit gene transcription, and elevated serum ST2 levels may be linked to inflammation and mortality. The IL-33 receptor is represented by its single ST2 receptor, which is expressed specifically by Th2 cells and is also called IL-33R or IL-RL1 [47]. The ST2 receptor is also expressed by inflammatory Th1 immune cells, Treg cells, and innate lymphoid cells (ILC), such as ILC2, ILC1, ILC2, ILC3, NK cells, and CD8^+^ T cells [48]. The “alarmin” IL-33 is secreted during angiogenesis by activated vascular endothelial and epithelial cells, including MCs, which express ST2 receptors [49]. This occurs in breast cancer, where MCs express the IL-33 receptor ST2. This cytokine activates CD34^+^ cells, contributing to MC differentiation and survival [50]. Moreover, this cytokine is expressed in fibroblast-like cells and is an important immunoregulator in both type 1 (inflammatory) and type 2 (allergic, autoimmune, and chronic diseases) immune responses. Therefore, human endothelial and epithelial cells constitute the major source of IL-33, which is expressed in many inflammatory disorders [49].

In addition, IL-33 is a pleiotropic cytokine that can activate ILC1 cells in immune reactions, such as tumor cell development [51]. ILC2s mediate tissue repair, immunological responses to allergens, immunotherapy, and cancer onset. However, IL-33 activates ILC2 cells, which, at the pathophysiological level, drive some human disorders, such as inflammation in asthma, allergy, atopic dermatitis, and fibrotic diseases.

Inflammation due to cytokines, such as IL-1, which activates IL-33, is inhibited by IL-37. The anti-tumor effect of IL-37 results from the suppression of tumor-promoting cytokines, such as IL-1β, which activates IL-6 and TNF, direct effects on tumor cells, and reduction of cell proliferation by interfering with NF-κB, MAPK, and signal transducer and activator of transcription 3 (STAT3). However, excessive cytokine gene silencing reduces immune surveillance. Low IL-37 levels may be associated with a worse prognosis, particularly in glandular cancers such as prostate cancer.

IL-38 is involved in the inhibition of inflammation, including tumor-related inflammation. It acts by blocking IL-36 and IL-1, thereby inhibiting tumor cell proliferation. IL-38 reduces the activity of Th17 and antigen-presenting dendritic cells. In experimental models, IL-38 inhibition enhances anti-tumor immunity by activating γδ T lymphocytes. Knockdown mice engineered with neutralizing antibodies or IL-38 gene silencing showed increased anti-tumor activity [52]. IL-38 or IL-37 may serve as prognostic biomarkers in certain cancers, such as prostate and breast cancer; however, there are currently no clinical therapies targeting IL-37 or IL-38. However, mechanistic analyses, such as co-immunoprecipitation for receptor complexes and RNA-seq, may be useful.

## 5. IL-37

More than 10 years ago, it was reported that inhibiting IL-37 causes an increase in pro-inflammatory cytokines in in vitro experiments on mononuclear cells [53]. Several studies have shown that tumor development is related to both innate and adaptive host immunity, where IL-1 family cytokines play a crucial role in the modulation of immune responses induced by IL-1 and toll-like receptors (TLRs) [54].

IL-37 is a natural cytokine that suppresses innate and adaptive immunity and inflammatory responses, and it is found at higher levels in inflammatory and autoimmune diseases, where it suppresses inflammation [55]. This cytokine is derived from the IL-18 subfamily, to which it is structurally closest, and in humans, the gene has been found on chromosome 2 [56]. IL-37 has five variants, IL-37a to IL-37e, with IL-37b being the largest, most functional, and most widely studied type [55]. The IL-37 gene is present in human cells, but not in mice, and upon activation, IL-37 is produced by various immune and non-immune cells. It binds to its receptor IL-18a/IL-1R8, inducing STAT3 and leading to pro-inflammatory polarization, and by inducing Akt and IKK, activates NF-κB, producing pro-inflammatory mediators [57]. IL-37 acts both intracellularly and extracellularly; extracellularly, it binds to the IL-18 receptor, but when it acts intracellularly, it migrates to the nucleus after being processed by caspase-1 and binds to TGF-b Smad3 [55].

**Figure 3:**
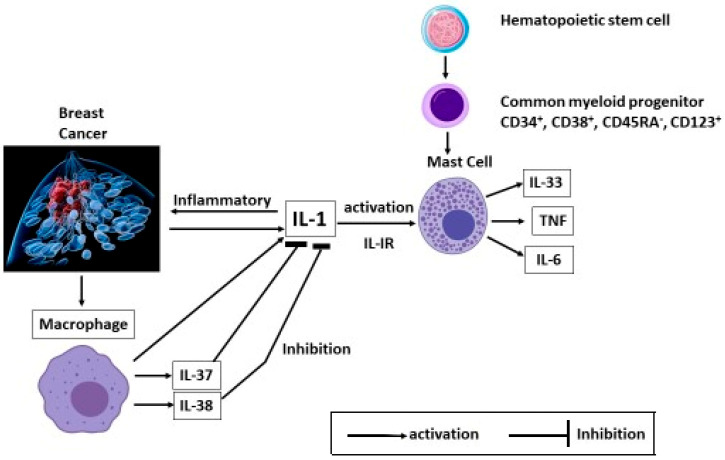
Signals released in the tumor microenvironment activate IL-1, which in turn produces inflammation in cancer tissues. IL-1 activates mature mast cells (MCs), which produce cytokines such as IL-6, tumor necrosis factor-α (TNF), and IL-33. Macrophages, after generating IL-1, produce IL-37 and IL-38 cytokines that suppress IL-1.

IL-37 is an immune molecule, mainly produced by monocytes, that almost completely inhibits the production of pro-inflammatory cytokines induced by IL-1 and/or activated TLR (Figure 3).

The role of IL-37 in cancer is complex and depends on the biological context, tumor type, immunosuppression, and inflammation status. IL-37 can be a friend in cancer by reducing tumor inflammation and suppressing NF-κB and MAPK pathways. IL-37 can be an enemy by causing excessive immunosuppression by inhibiting T cell and dendritic cell maturation. High levels of IL-37 are associated with more aggressive tumors and worse prognosis.

In experiments on transgenic mice expressing human IL-37 genes, it was noted that these animals reacted better after bacterial endotoxin inoculation and presented less severe organ lesions than wild-type mice. IL-37 protects against aging, cardiovascular disease, and other inflammatory diseases [55]. Moreover, transgenic mice possessing human IL-37 genes have the ability to reject tumor cells, and overexpression of this cytokine in some tumors leads to better overall survival [25]. In experiments on mice, Gao et al. found that by treating these rodents with intratumor doses of IL-37, they achieved a reduction in tumor activity and cancer cell growth [58]. Furthermore, experiments on transgenic mice have shown that IL-37 protects against LPS-induced septic shock and stimulates adaptive immunity by acting on regulatory Treg and dendritic cells [58].

IL-37 can favor tumor activity by inducing immune tolerance, whereas it can counteract tumor development and growth by suppressing inflammation. Thus, for breast cancer, this anti-inflammatory and immunosuppressive cytokine could be of great interest and may be a candidate for immunotherapy. However, it is important to understand whether IL-37 is a friend or foe of cancer.

IL-37 is an anti-inflammatory cytokine that suppresses inflammation and plays a complex role in oncology, depending on the biological context. In some cases, they can both promote and antagonize tumor activity. IL-37 suppresses many pro-inflammatory pathways, including NF-κB, MAPK, and the cytokines IL-1β, IL-6, and TNF, thereby inhibiting tumor growth. However, these inflammatory pathways may be necessary to support the activation of dendritic cells, T lymphocytes, anti-tumor NK cells, and the TH1/CTL response, which are important for the immune response [59].

The investigation of IL-37 in human tumors is quite limited, and its role requires further study. Moreover, the literature has reported conflicting data on this cytokine, and the role of its biological activity in cancer remains to be defined.

IL-38 is another anti-inflammatory cytokine produced by macrophages that inhibits inflammation.

## 6. IL-38

IL-38, also called IL-1F10, is the most recently discovered cytokine of the IL-1 family, encoded on the X chromosome, and plays a crucial role in inflammatory diseases [60].

IL-38 exerts its biological activity by binding to its receptor IL-36R6 and provoking an anti-inflammatory effect. It inhibits several inflammatory cytokines, including IL-1 and IL-6 [61]. At the intracellular level, IL-38 exists as a precursor that must be cleaved at the N-terminus and secreted at the extracellular level in an active form with anti-inflammatory activity [39]. In addition, IL-38 inhibits several chemokines, such as macrophage inflammatory protein alpha (MIP-3α) (termed CCL20) and IL-8 (also termed CXCL8), which induce neutrophil chemotaxis through its type 1 receptor (IL-8R1) [39].

Blocking IL-8 limits neutrophil influx in breast cancer, reducing the inflammatory state that favors tumor development and its pathological state [62]. The inhibitory effects of IL-8 are important, as this chemokine is also chemotactic for endothelial cells, which play a key role in angiogenesis. IL-8 acts on phospatidylinositol-3-kinase (PI3K) and MAPK, inducing the phosphorylation of Akt, which plays a key role in angiogenesis, cell migration, and metastasis, contributing to tumor development and cancer stem cells [63]. Therefore, it is conceivable that antagonizing the IL-8 receptor could be useful for breast cancer therapy [64]. MIP-3a is also a chemokine that promotes cancer progression by affecting tumor cell migration and proliferation through immune cell control [65].

The IL-1 family includes the IL-36 subfamily, consisting of IL-36α, IL-36β, IL-36γ, and IL-38, which share the same IL-1R6 receptor [66]. The inflammatory action of the cytokine IL-36 is specifically inhibited by IL-36 receptor antagonists (IL-36Ra). IL-38 is a naturally occurring anti-inflammatory and anti-cancer cytokine that inhibits IL-1 and suppresses its biological activity by binding to the IL-1R6 receptor [60]. It inhibits the generation of IL-17, a cytokine composed of IL-17A, B, C, D, E, and F, which, by binding to the respective receptors and through NF-κB and MAPKs, leads to the activation of chemokines, cytokines, and anti-inflammatory genes [67].

There are experimental in vitro and some preclinical data showing that IL-38 can reduce the production of IL-8 and Th17 cytokines (including IL-17/IL-22). However, there is still no direct and specific evidence that IL-38 inhibits IL-8 and IL-17 expression within the breast cancer microenvironment. Some studies have shown the release and role of IL-38 in breast cancer cells (e.g., MDA-MB-231), and more recent preclinical studies have indicated that modulating IL-38 alters anti-tumor immunity [68]. IL-38 and some truncated forms suppress IL-8 secretion, especially when induced by IL-36γ. Mora et al. documented that tumor lines (including the MDA-MB-231 breast cancer cell line) release IL-38 following apoptosis; IL-38 present in apoptotic cell-conditioned media affects macrophage responses and cytokine profiles [69]. Modulating IL-38 may change the balance of pro-/anti-inflammatory cytokines; however, the specific effects of IL-8/IL-17 in breast cancer remain to be characterized [70].

Along with other cytokines, IL-17 is generated by activated cells such as T, Th17, CD8^+^, memory, and naïve CD4^+^ T cells [71]. By producing IL-21, these cells activate STAT3, with subsequent production of IL-23, a cytokine that can induce Th17 cells to produce IL-17 [72]. In addition, as IL-17/Th17 is associated with autoimmune diseases, inhibiting this cytokine could represent a new therapeutic strategy. However, the involvement of Th17 cells in tumor diseases is still not well understood, as on the one hand, they may be associated with anti-tumor responses, while on the other hand, they may play a critical role in the growth, proliferation, inflammation, and invasion of tumor cells.

Much of the data on IL-37 and IL-38 comes from in vitro models or preclinical studies on various tumors; however, specific experimental data on breast cancer are scarce or insufficient and are still under investigation [39]. In our laboratory, we are preparing a list of practical experiments that could be performed to directly test both IL-37 and IL-38 in breast cancer models (tumor cells, co-cultures with macrophages and lymphocytes) using these recombinant anti-inflammatory cytokines. IL-37 suppresses excessive immune responses in various tumors and acts as an antiproliferative factor by inhibiting NF-κB and reducing pro-inflammatory cytokines. IL-38 is an anti-inflammatory cytokine that regulates immune pathways related to IL-36R and inflammasomes. It regulates the tumor microenvironment primarily by reducing pro-inflammatory cytokines and the infiltration of pro-tumor immune cells. IL-37 acts by forming a complex with IL-18Rα (IL-1R5) and IL-1R8 (SIGIRR), a reaction that is important for its anti-inflammatory signals, while IL-38 is a more complex molecule that often functions as an antagonist of IL-36 signaling (binding IL-36R and other receptors) and can promote tumor immunosuppression. Indeed, in experimental models, IL-38 inhibition enhances anti-tumor immunity by activating γδ T lymphocytes [52]. However, IL-37 and IL-38 are not yet approved by the FDA (Federal Drug Administration).

IL-37 transgenic mice (*IL-37tg*) show a reduction in pro-inflammatory cytokines and inhibition of tumor growth and metastasis. In vitro and in vivo experiments using recombinant human IL-37 have been performed to evaluate its effects on angiogenesis, apoptosis, and sensitivity to chemotherapy [73]. Low IL-37 levels may be associated with a worse prognosis, particularly in glandular cancers such as prostate cancer.

Furthermore, IL-38 inhibits IL-22 in vitro by human peripheral blood mononuclear cells (PBMCs), which are cytokines secreted by Th22 cells activated by CD4^+^ naïve T cells after antigen presentation by dendritic cells or macrophages [74]. IL-22 is involved in breast cancer cell proliferation, which is a STAT3-dependent effect [75]. Th17 cells intervene in breast cancer and create an inflammatory environment through the production of cytokines, including IL-22, which could help fight tumor development [76]. Th17 cells have a certain plasticity and can transform into Th2, Treg, and/or Th1 cells, showing contrasting functions depending on the environment in which they are activated [77]. Th2 cells can play a pathological role, such as in asthma, while Tregs can regulate the immune system in autoimmune diseases and breast cancer.

In cancer, activated dendritic cells trigger naïve T cells through the T cell receptor to release TGF-β and IL-6, which activate Th17 [78] to release IL-23 that binds IL-23R and, in an autocrine loop manner, stimulates effector or memory Th17 cells to secrete IL-17, IL-17F, and IL-22 [79]. Moreover, IL-38 reduces the secretion of IL-23 in mice, and since ILC3 cells produce IL-22, IL-23, and other cytokines, their inhibition could reduce breast cancer development [80].

It has been hypothesized that IL-38 exerts its anti-inflammatory action by binding to its receptor, IL-1R9. This cytokine was found to be elevated in inflammatory diseases, including cardiovascular events, as a counteracting response of the body to inflammation [81]. IL-38 is mainly produced by B cells and macrophages and is found in high concentrations in the synovium of patients with rheumatoid arthritis [82]. In fact, rodents with induced inflamed joints exhibit elevated levels of IL-38, and deficiency of this cytokine results in overexpression of pro-inflammatory cytokines [83]. In addition, we recently reported that IL-38 inhibits the inflammatory molecules IL-1b and CXCL8 in microglia stimulated by the neuropeptide neurotensin [81]. Therefore, this cytokine inhibits the immune system and, like IL-37, inhibits the gene expression of IL-1b and other cytokines and chemokines, including CXCL8.

IL-38 is also increased in autoimmune diseases compared to healthy subjects, and IL-38 reduces several pathological signs when used in treatments for patients [84]. In cardiovascular disease, IL-38 inhibits inflammation and calcium deposition in aortic valve interstitial cells by inhibiting caspase-1, IL-1, intercellular adhesion molecule-1 (ICAM-1), and vascular cell adhesion molecule-1 (VCAM-1) [85].

In conclusion, we report that MCs surround the breast tumor stroma by releasing mediators that promote tumor cell growth and development and molecules that inhibit cancer and metastasis. Additionally, we hypothesized that blocking pro-inflammatory cytokines, including IL-1, could be a novel therapeutic strategy against inflammatory diseases, including breast cancer. Therefore, MCs in breast tumor tissue can have both positive effects by producing anti-cancer molecules and negative effects by promoting tumor growth.

## Figures and Tables

**Table 1:** The relationship between tumors and mast cells (MCs): Effect of IL-37 and IL-38. The interaction between tumors and MCs leads to the production of mediators that cause MC recruitment and activation. This interaction causes mast cells to release various compounds, including vascular endothelial growth factor (VEGF), tryptase, IL-10, and transforming growth factor-β (TGF-β), which promote tumor growth and immunosuppression. IL-37 inhibits the production of histamine and proteases in MCs, which can block pro-angiogenic mediators. IL-38 inhibits NF-κB, resulting in the inhibition of the production of inflammatory cytokines.

Interaction	Production	Cause
Tumor/Mast Cell	SCF/*c-Kit*, CXCL12, IL-33	Mast cell recruitment and activation
Mast Cell/Tumor	vascular endothelial growth factor (VEGF), tryptase, IL-10, transforming growth factor-β (TGF-β)	Tumor growth and immunosuppression
IL-37/Mast Cell	Inhibits histamine and proteases	Blocks pro-angiogenic mediators
IL-38/Mast Cell	Reduces NF-κB	Inhibition of inflammatory cytokines
IL-37/IL-38/Tumor	Decreases inflammation	angiogenesis, proliferation

**Table 2:** Compounds released from mast cells (MCs) upon activation can affect tumor cell growth.

**Immediately Released:**	Carboxypeptidase A, Cathepsin G, Cholinesterase, Chymase, Histamine, Kininogenase, TNF, Tryptase
**De Novo Synthesis:**	IL-1, 2, 3, 4, 5, 6, 10, 11, 13, IL-33, IFNγ, TNF, NO, VEGF, PGD2, LTB4, LTC4, CXCL8, CCL2, CCL7, MCP-4, CCL5, CCL10, CCL9, CCL11, CCL20, PAF

**Table 3:** Mast cells (MCs) can be activated by various factors, including cytokines involved in tumor growth.

**Environmental:**	Virus, Bacteria, Fungi, lipopolysaccharide (LPS), Parasites, Venoms, Mercury
**Drugs:**	Adenosine, Estradiol, Morphine, Non-steroidal anti-inflammatory drugs (NSAIDs)
**Peptides:**	Corticotropin-releasing hormone (CRH), Endothelin, Adrenomedullin, Endorphin, Neurotensin, Substance P, Vasoactive intestinal peptide (VIP), Thrombin, *c-Kit*, IgG, IgE
**Cytokines and Growth Factor:**	IL-1, 4, 6, 33, Nerve Growth Factor (NGF), Stem Cell Factor (SCF)

## Data Availability

No new data were created or analyzed during this study. Data sharing is not applicable to this article.

## Abbreviations

APC	antigen-presenting cell
APP	acute phase protein
CRH	corticotropin-releasing hormone
CSF	colony-stimulating factor
CTL	cytotoxic T cell
ERK	extracellular signal-regulated kinase
G-CSF	granulocyte colony-stimulating factor
GM-CSF	granulocyte-macrophage colony-stimulating factor
ICAM-1	intercellular adhesion molecule-1
IFN-g	interferon gamma
IL-1RAcP	co-receptor accessory protein
ILC1	type 1 innate lymphoid cells
ILC2	type 2 innate lymphoid cell
ILC3	type 3 innate lymphoid cell
LPS	lipopolysaccharide
MAPK	mitogen-activated protein kinase
MC	mast cell
MCP-1/CCL2	monocyte chemoattractant protein-1
MIP-3α	macrophage inflammatory protein alpha
NF-κB	nuclear factor kappa B
NGF	nerve growth factor
NK	natural killer
NLRP3	NOD-like receptor family pyrin domain containing 3
NO	nitric oxide
NSAID	non-Steroidal anti inflammatory drug
PAMP	pathogen-associated molecular pattern
PD-L1	programmed death-ligand 1
PDGF	platelet-derived growth factor
PI3K	phospatidylinositol-3-kinase
RANTES	regulated on activation, normal T cell expressed and secreted
ROS	reactive oxygen species
SAA	serum amyloid A
SCF	stem cell factor
ST2/IL-1R4	suppression of tumorgenicity 2
STAT3	signal transducer and activator of transcription 3
TGF-β	transforming growth factor-β
Th	T helper cell
TLR	Toll-like receptor
TNF	tumor necrosis factor
Treg	T regulatory
VCAM-1	vascular cell adhesion molecule-1
VEGF	vascular endothelial growth factor
VIP	vasoactive intestinal peptide
